# Heritability of Age-Related Macular Degeneration in the Amish

**DOI:** 10.64898/2026.08.04.26359695

**Published:** 2026-08-06

**Authors:** Noel C. Moore, Yeunjoo E Song, Alexander V. Gulyayev, Kristy Miskimen, Penelope Miron, Renee A Laux, Audrey Lynn, Sarada L Fuzzell, Sherri D Hochstetler, Dawn Miller, Laura J Caywood, Jason E Clouse, Sharlene D Herington, Ping Wang, Yining Liu, Daniel A Dorfsman, Jeffery M Vance, Muneeswar Gupta Nittala, SriniVas R Sadda, Dwight Stambolian, William K Scott, Margaret A Pericak-Vance, Jonathan L Haines

## Abstract

**Purpose:**

Age-related Macular Degeneration (AMD), a degenerative disease of aging, leads to central vision loss and has a strong genetic risk. Genetic heritability, used to quantify genetic influence on a trait, has mainly focused on twin study designs but these are vulnerable to bias. Studying relatives beyond twins is necessary to bring clarity to the genetic burden of AMD and help focus the search for additional genetic risk loci.

**Methods:**

Through both single nucleotide polymorphism (SNP) and pedigree-based heritability methods, the heritability of AMD was analyzed using relationship informed analyses of families from an Amish population (n = 525). AMD status was determined using the Beckman grading scale (285 controls and 240 cases). An estimate of genetic relatedness preceded SNP heritability estimation, whereas the pedigree heritability model utilized genealogical reports. Primary models were adjusted for age, sex, and population structure. A comparison of SNP- and pedigree-based models followed heritability estimation. Sensitivity models adjusting for all possible combinations of three known strong AMD genetic risk variants were constructed.

**Results:**

SNP heritability is 55% +/- 13% (p= 9.87e-06) and the pedigree heritability is 49% +/- 18% (p= 3.06e-04). The sensitivity analyses revealed that the estimates were robust to changes in the inclusion of AMD variants as covariates.

**Conclusions:**

These heritability estimates support existing twin and SNP-based AMD heritability estimates and corroborate the substantial involvement of genetics in AMD. Adjusting for known AMD variants revealed that additional genetic contribution exists, supporting a large polygenic effect in AMD.

## Full Text Availability

The license terms selected by the author(s) for this preprint version do not permit archiving in PMC. The full text is available from the preprint server.

